# Clinical Outcomes After Valve Intervention in Rheumatic Mitral Valve Disease

**DOI:** 10.5334/gh.1420

**Published:** 2025-04-04

**Authors:** Marco Aurélio da Silva Neves, Lucas Leal Fraga, Moises Barbosa de Andrade, Bruno Ramos Nascimento, Cláudio Leo Gelape, Renato Bráulio, Paulo Henrique Nogueira Costa, Marcia Fabrícia Almeida Teixeira, Paulo Henrique Moreira Melo, Guilherme Rafael Sant’Anna Athayde, Lucas Lodi-Junqueira, Robert A. Levine, Maria Carmo Pereira Nunes

**Affiliations:** 1 Programa de Pós-Graduação em Ciências da Saúde: Infectologia e Medicina Tropical da Universidade Federal de Minas Gerais, Belo Horizonte, MG, Brazil; 2 Hospital das Clínicas da Universidade Federal de Minas Gerais, Belo Horizonte, MG, Brazil; 3 Division of Cardiology, Department of Medicine, Massachusetts General Hospital, Harvard Medical School, Boston, MA, USA

**Keywords:** Rheumatic heart disease, mitral valve disease, percutaneous mitral valvuloplasty, cardiac surgery, mitral valve replacement

## Abstract

**Background:**

Rheumatic heart disease (RHD) remains one of the leading causes of mitral valve (MV) disease in developing countries. Despite the availability of percutaneous and surgical interventions, long-term outcomes remain unclear. This study aims to identify determinants of outcomes following percutaneous or surgical intervention in patients with rheumatic MV disease, addressing critical gaps in treatment selection.

**Methods:**

A retrospective, intention-to-treat study was conducted on patients with symptomatic rheumatic MV disease, primarily characterized by mitral stenosis, who underwent either percutaneous mitral valvuloplasty (PMV) or MV replacement (MVR). Demographic, clinical, and echocardiographic variables were collected. The long-term outcome was defined as a composite of death, repeat PMV, need for cardiac surgery, and stroke.

**Results:**

A total of 246 patients were enrolled (mean age 43.8 ± 13 years, 80% women, with 45% in New York Heart Association [NYHA] class III/IV). Of these, 90 patients (37%) underwent MVR, while 156 patients (63%) underwent PMV, with similar clinical characteristics at baseline. During a mean follow-up of 2.8 years, ranging from 1 day to 7.8 years, 45 patients (18%) reached the composite outcome, including 11 deaths (4%). Long-term outcomes were comparable between PMV and MVR (P = 0.231). Independent predictors of composite outcomes included baseline NYHA class III/IV (adjusted hazard ratio [HR] 2.10, 95% confidence interval [CI] 1.10–4.11, P = 0.023) and older age (HR 1.03, 95% CI 1.01–1.06, p = 0.020). Predictors of all-cause mortality following either PMV or MVR were older age (HR 1.08, 95% CI 1.03–1.14, P = 0.002) and lower left ventricular ejection fraction (HR 0.93, 95% CI 0.88–0.99, P = 0.021).

**Conclusions:**

This study identified older age and higher NYHA functional class as significant predictors of composite outcomes in patients with rheumatic MV disease requiring intervention. Left ventricular systolic dysfunction was independently associated with increased mortality following both percutaneous and surgical intervention. Long-term outcomes were comparable between patients undergoing PMV and MVR, reinforcing PMV as an effective alternative to surgery in appropriately selected patients.

## Introduction

Rheumatic heart disease (RHD) remains the leading cause of valvular heart disease in children and young adults worldwide, contributing to significant morbidity and mortality (1,2). The mitral valve (MV) is the most commonly affected, with a substantial proportion of patients presenting with mixed MV disease rather than isolated stenosis or regurgitation (3,4,5,6,7).

While percutaneous mitral valvuloplasty (PMV) is the preferred intervention for isolated or predominant mitral stenosis (MS), and surgery is recommended for patients with significant mitral regurgitation (MR) or unfavorable valve morphology, the optimal management approach remains complex (8,9,10,11). Clinical decision-making must balance patient-specific factors, procedural risks, and local expertise (8,9,12). Notably, disparities in access to interventional therapies persist, particularly in low- and middle-income countries (LMICs), where many patients who require intervention do not receive timely treatment (1,13).

Although PMV has demonstrated outcomes comparable to open mitral commissurotomy for MS, the choice between surgical valve repair and replacement in rheumatic MV disease remains a subject of debate (14,15). While MV repair reduces prosthetic-related complications, it carries a higher risk of reoperation (16). Given the evolving landscape of treatment strategies, further investigation is needed to refine patient selection criteria and optimize long-term outcomes for patients with rheumatic MV disease (17,18,19,20).

Therefore, this study aims to: 1) characterize the pattern of rheumatic valve involvement in patients referred for MV intervention at our institution; 2) compare the clinical and echocardiographic characteristics of patients based on the type of valve intervention; 3) identify factors associated with adverse outcomes following percutaneous or surgical intervention; and 4) assess potential differences in long-term event-free survival between PMV and MVR in this population.

## Material and Methods

### Study Population

This retrospective study included patients with symptomatic rheumatic MV disease who were referred for valve intervention—either PMV or MVR—between January 2010 and September 2016 at Hospital das Clínicas, Universidade Federal de Minas Gerais, Brazil.

### Inclusion and exclusion criteria

Patients were included if they met the following criteria:

- Confirmed rheumatic MV disease based on echocardiographic morphological features, including leaflet thickening, restricted leaflet mobility, commissural fusion, chordal thickening, and shortening (21,22).
- Severe MV disease, as defined by current guideline recommendations for intervention (8,9,11). Severe MS was defined as a mitral valve area ≤1.5 cm² and significant MR was defined as moderate or severe MR, according to established echocardiographic grading systems (23),
- Symptomatic status, requiring intervention based on dyspnea (NYHA class II-IV) or pulmonary hypertension.

Exclusion criteria included patients with prior valve prosthesis dysfunction undergoing cardiac surgery for prosthetic valve replacement.

PMV was performed in symptomatic patients with severe MS and favorable valve morphology, excluding those with significant MR. Surgery was performed in patients with combined MV disease, predominant MR, or for patients with MS who were unsuitable for percutaneous procedures or who have failed previous PMV (8). Patients who had undergone prior PMV were eligible for repeat PMV or MVR based on their clinical and echocardiographic characteristics.

Patients with associated severe aortic stenosis or regurgitation were referred for surgery, whereas those with moderate aortic involvement underwent either MVR or PMV according to the predominant MV disease, based on symptoms and left ventricular remodeling.

Given the retrospective nature of the study, the sample size was determined by the number of patients meeting the inclusion criteria within the study period. While retrospective studies may introduce selection bias, our approach aimed to minimize this by including all consecutive patients referred for intervention during the study period. Additionally, adjustments for potential confounders were applied in the statistical analysis.

The study protocol was approved by the institutional review board of Universidade Federal de Minas Gerais, Brazil.

### Echocardiography

A standard transthoracic echocardiogram was performed following the American Society of Echocardiography guidelines (23). Mitral valve morphology was evaluated according to recommendations, taking into account valve thickening, mobility, calcification, and subvalvular chordal fusion, as well as the assessment of commissural morphology (10,24,25). Peak and mean transmitral diastolic pressure gradients were measured from pulsed-wave Doppler profiles recorded in the apical four-chamber view. The presence and severity of MR were evaluated according to guidelines (23). The tricuspid regurgitant velocity was recorded with continuous-wave Doppler imaging and used to determine the systolic pulmonary artery pressure using the modified Bernoulli equation.

### Mitral valve interventions: surgical and percutaneous mitral valve procedure

MVR was performed with complete preservation of the subvalvular apparatus. The choice of prosthetic valve and suture technique was left to the surgeon’s discretion.

PMV was performed using an anterograde trans-septal approach using the Inoue balloon technique, as previously described (26). Procedural success was defined as a post-procedure MV area was ≥1.5 cm^2^ without a significant increase in MR (25).

### Study endpoint definitions

The primary outcome was a composite of long-term adverse events, including all-cause mortality, cardiovascular-related death, mitral valve replacement, repeat PMV, or stroke. Cardiovascular mortality was defined as death due to stroke, heart failure, sudden death, or procedure-related mortality. Other deaths were considered non-cardiovascular.

Patients who underwent unsuccessful PMV requiring subsequent MVR were analyzed in the PMV group (intention-to-treat principle). Outcome data were collected through clinic follow-ups and telephone interviews with patients or their relatives.

### Statistical analysis

Categorical data were presented as numbers and percentages and compared using the Chi-square test. Continuous variables were expressed as mean ± standard deviation (SD) or median and interquartile range (IQR), depending on the distribution, and compared using the unpaired Student’s t-test or Mann-Whitney test as appropriate.

Cox proportional hazards regression analyses were performed to identify independent predictors of mortality and composite outcomes. In the univariable Cox hazard model, we included all relevant variables, and those statistical significances were entered into a multivariable model. To address potential confounding, variables were assessed for collinearity, and redundant or highly interdependent covariates were excluded from simultaneous inclusion in the analyses. The final multivariable model was then adjusted for baseline clinical and echocardiographic characteristics that could act as potential confounders.

As missing values were <2% for key variables, the final model was based on complete data, and no imputation techniques were applied.

Kaplan-Meier curves were constructed for all-cause mortality and composite outcomes and compared using the log-rank test.

A P-value <0.05 was considered statistically significant. Statistical analyses were conducted using SPSS version 18 (SPSS Inc., Chicago, IL).

## Results

### Baseline Characteristics of the Study Population

**Figure 1:**
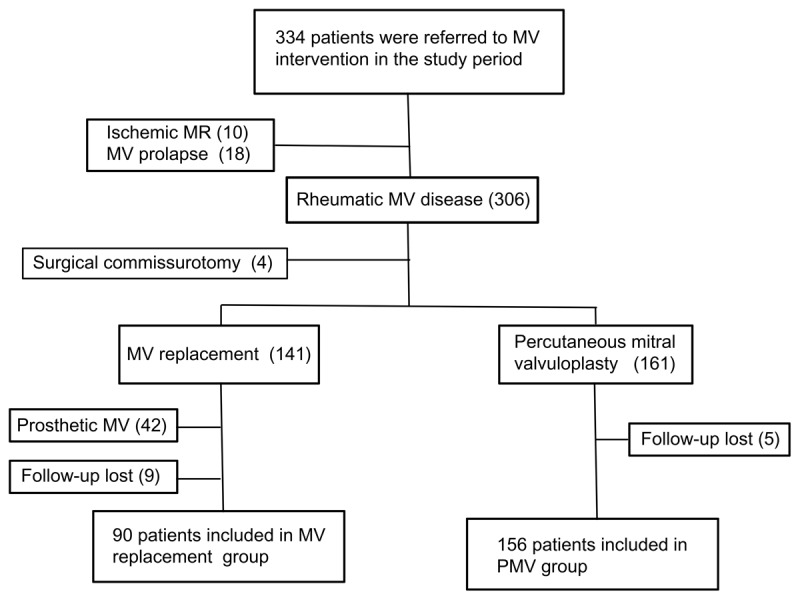
Flow diagram. Of 334 patients who underwent mitral valve replacement or percutaneous mitral valvuloplasty, 302 with rheumatic MV disease were initially eligible for the study. Forty-two patients who had prosthetic MV were excluded. Additionally, 14 patients were lost to follow-up, leaving 246 with outcomes data available for analysis.

**Figure 2:**
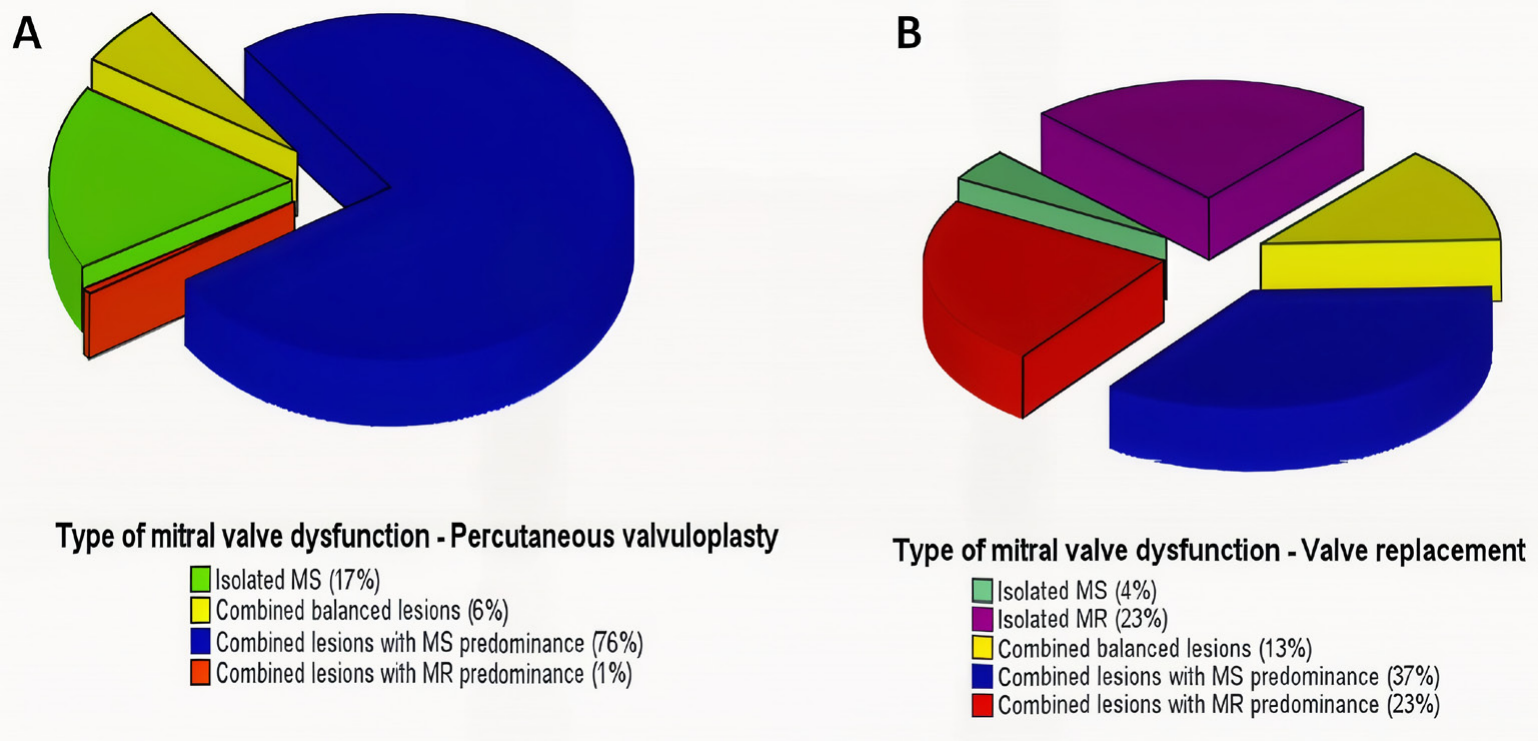
A: Type of mitral valve dysfunction in patients undergoing percutaneous mitral valvuloplasty. **B:** Type of mitral valve dysfunction in patients undergoing surgical mitral valve replacement.

Of the 334 patients who underwent mitral valve intervention, 302 had rheumatic MV disease. Forty-two patients who had prosthetic MV were excluded, and 14 patients were lost to follow-up, leaving 246 with complete outcome data for analysis. Ninety patients underwent MVR, whereas 156 underwent PMV (Figure 1). The mean age was 43.8 ± 12.9 years, and 198 (80%) were women. The types of MV dysfunction according to the procedure are shown in Figure 2A and 2B.

Among patients referred to PMV, the most frequent valve dysfunction was combined MS and regurgitation with predominance of stenosis (119 patients, 76%), whereas isolated MS was found in 27 patients (17%). Patients with combined balanced lesions who underwent PMV had the symptoms related to MS, without indications for surgery to treat the regurgitation lesion. A single patient with predominance of MR, deemed unsuitable for surgery secondary to cancer, underwent PMV for relief of pulmonary congestion (Figure 2A).

The predominant valvular lesion in the patients who underwent surgery was combined MS and MR with predominance of stenosis (33 patients, 37%), followed by combined balanced lesions (32 patients, 36%). Isolated MR was detected in 21 patients (23%) and isolated MS without any degree of regurgitation in only 4 patients (4%) (Figure 2B).

The baseline clinical characteristics of the study population stratified by the type of MV intervention are summarized in Table 1. Patients were similar regarding age and NYHA functional class. The PMV group had a predominance of women, whereas atrial fibrillation was more frequent in the surgical group.

**Table 1:** Baseline characteristics of patients with rheumatic mitral valve disease who underwent percutaneous mitral valvuloplasty (PMV) or surgical mitral valve replacement (MVR).


VARIABLES*		PERCUTANEOUS VALVULOPLASTY* (n = 156)	SURGICAL VALVE REPLACEMENT (n = 90)	P-VALUE

Age (years)		42.7 ± 12.1	45.6 ± 14.3	0.095

Body surface area (m^2^)		1.67 ± 0.2	1.66 ± 0.2	0.835

Gender	Female	138 (88)	60 (67)	<0.001

	Male	18 (12)	30 (33)	

NYHA funcional class	I/II	87 (56)	49 (54)	0.676

	III/IV	69 (44)	41 (46)	

Previous valvuloplasty^†^		59 (38)	13 (14)	<0.001

Atrial fibrillation		32 (21)	34 (38)	0.003

Hypertension		38 (42)	44 (28)	0.025

Diabetes		5 (3)	7 (8)	0.109

Coronary artery disease		2 (1)	2 (2)	0.574

Previous stroke		20 (13)	11 (12)	0.892

Heart rate (bpm)		73.8 ± 12.6	76.4 ± 11.9	0.159

Systolic blood pressure (mmHg)		115.7 ± 16.3	120.1 ± 18.4	0.111

Diastolic blood pressure (mmHg)		74.1 ± 10.2	73.0 ± 11.2	0.547

**Medication**				

Diuretics		101 (65)	78 (87)	0.001

Beta-blockers		106 (68)	65 (72)	0.550

ACE inhibitors		36 (23)	45 (50)	<0.001

Anticoagulants		41 (26)	25 (28)	0.709


* Values are expressed as the mean value ± SD, or absolute numbers (percentage). † Mitral valvuloplasty included either percutaneous (n = 42) or surgical intervention (n = 26) or both (4). ACE: angiotensin-converting-enzyme.

Similarly, use of diuretics and angiotensin-converting-enzyme inhibitors was more frequent in the surgical group. Mitral valvuloplasty had previously been performed in 72 patients (29%), including either percutaneous or surgical intervention. Patients who were referred for PMV had more frequent previous valvuloplasty than those referred to MVR.

### Echocardiographic features

Echocardiographic parameters in the two groups are presented in Table 2. The left ventricular ejection fraction was slightly higher in the MVR group. The largest difference between the groups was in left ventricular diameters. The patients in the surgical group presented with larger end-diastolic and end-systolic dimensions compared to the PMV group. Severe MS defined by valve area less than 1 cm^2^ was found in 98 patients (63%) and moderate (valve area between 1–1.5 cm^2^) in 58 patients (37%) of the PMV group. In the MVR group, severe MS was found in 47 patients (52%), moderate in 19 (21%), mild (area more than 1.5 cm^2^) in 17 patients (19%), and no MS in 7 cases (8%). Regarding the degree of MR, moderate and severe MR was observed in 6 patients (4%) in the PMV group compared with 67 patients (74%) in the MVR group.

**Table 2:** Echocardiographic features of patients with rheumatic mitral valve disease stratified according to the type of valve intervention.


	PERCUTANEOUS VALVULOPLASTY* (n = 156)	SURGICAL VALVE REPLACEMENT (n = 90)	P VALUE

LA diameter (mm)	49.9 ± 6.1	56.3 ± 9.9	**<0.001**

LVDd (mm)	48.1 ± 6.1	55.9 ± 8.8	**<0.001**

LVSd (mm)	31.1 ± 4.9	38.0 ± 7.2	**<0.001**

LVEF (%)	63.8 ± 7.3	60.2 ± 9.6	**0.003**

Mean gradient (mmHg)	11.1 ± 5.2	11.3 ± 5.7	0.768

Mitral valve area (cm^2^)^†^	1.0 ± 0.3	1.3 ± 0.6	**<0.001**

Moderate/severe TR	32 (20)	28 (31)	0.055

Moderate/severe MR	6 (4)	67 (74)	<0.001

SPAP (mmHg)	51.4 ± 18.3	50.2 ± 16.5	0.661

Echocardiographic score	7.0 ± 1.3	10.0 ± 1.9	**<0.001**


* Patients who underwent surgical valvuloplasty (commissurotomy) instead of surgical valve replacement during the time of the study were not included. † Mitral valve area was calculated by planimetry. LA = left atrium; LVDd = left ventricular end-diastolic diameter; LVEF = left ventricular ejection fraction; LVSd = left ventricular end-systolic diameter; MR = mitral regurgitation; SPAP = systolic pulmonary artery pressure; TR = tricuspid regurgitation.

As expected, echocardiographic Wilkins’ score was lower in the patients who underwent PMV. Systolic pulmonary artery pressure and mean transmitral gradients were similar between the groups.

Functional tricuspid regurgitation associated with MV lesions was found in 60 patients (24%). Rheumatic aortic valve involvement was frequent in this population, with 124 patients (50%) presenting some degree of aortic valve disease, usually mild aortic regurgitation (39%). Fifty-two patients had associated aortic stenosis (21%) with a mean valve area of 1.22 cm^2^ (range 0.8–1.6 cm^2^).

The surgical procedure for MVR was combined with aortic valve replacement in 14 patients (16%), correction of functional tricuspid regurgitation with annuloplasty in 7 (8%), and coronary artery bypass surgery in 2 (2%).

### Long-term outcomes

The overall mean follow-up time was 2.8 years (range 1 day–7.8 years); the median was 3.2 years for the surgical group and 2.7 years for the valvuloplasty group (p = 0.08). During the follow-up period, 45 adverse clinical events were observed. Twelve patients died (2 non-cardiac deaths), 22 patients underwent MVR, and 7 repeated PMV. Additionally, 4 patients had a stroke. The outcomes according to the type of valve intervention are shown in Table 4. The mortality was higher in the MVR group, whereas the need for valve intervention (repeated PMV and valve replacement) was higher in the PMV group.

### Outcomes in PMV group

PMV resulted in adequate valve opening without an increase in MR or other complications in 131 patients (84%). The procedure was considered unsuccessful in 25 patients (16%), due to insufficient valve opening in 8 patients (5%) or a greater than one-grade increase in MR grade in 17 patients (11%). Additionally, 6 patients (4%) required emergency MVR surgery due to severe MR following PMV. During a median follow-up of 2.7 years, 31 adverse clinical events were observed, including 3 deaths, 20 mitral valve replacements, 7 repeat PMVs, and 1 stroke (Table 3). Long-term outcome after PMV was strongly determined by the quality of immediate procedural results (hazard ratio (HR) 3.352; 95% CI 2.396–4.690, p < 0.001).

**Table 3:** Clinical endpoints according to the type of valve intervention.


	PERCUTANEOUS VALVULOPLASTY* (n = 156)	SURGICAL VALVE REPLACEMENT (n = 90)	P VALUE

All-cause mortality	3 (1.9)	9 (10)	

Cardiovascular death	3 (1.9)	7 (7.8)	

MV replacement	20 (12.8)	2 (2.2)	<0.001

Repeat PMV	7 (4.5)	0	

Stroke	1 (0.6)	3 (3.3)	


* Values are expressed as absolute numbers (percentage). MV = mitral valve; PMV = percutaneous mitral valvuloplasty.

### Outcomes in surgical group

In the MVR group, there were 3 operative deaths. Eleven patients required reoperation due to cardiac tamponade (n = 8), major bleeding (n = 2), and mediastinitis (n = 1). Additionally, nine patients presented with postoperative infections. During a mean follow-up of 3.2 years, 14 adverse events were observed, including 9 deaths (2 non-cardiac deaths), 2 needed further valve replacement, and 3 strokes (Table 3).

### Predictors of long-term outcome

Univariable Cox proportional-hazards analysis for predicting all-cause mortality and the composite outcome are shown in Table 4. Clinical and echocardiographic parameters that express the severity of MV disease or its hemodynamic consequences were tested in the univariable analysis.

**Table 4:** Univariable Cox proportional-hazards analysis for predicting cardiac death and composite outcome.


VARIABLES	ALL-CAUSE MORTALITY		COMPOSITE OUTCOME	
	
	HR (95% CI)	p-VALUE	HR (95% CI)	p-VALUE

PMV	0.303 (0.105–0.875)	0.027	1.473 (0.782–2.777)	0.231

Age (years)	1.063 (1.020–1.107)	0.004	1.024 (1.001–1.049)	0.045

NYHA class III/IV	2.144 (1.075–4.275)	0.030	2.661 (1.415–5.006)	0.002

Atrial fibrillation	4.201 (1.523–11.589)	0.006	1.383 (0.750–2.551)	0.299

LVEF (%)	0.926 (0.880–0.975)	0.004	0.980 (0.946–1.014)	0.246

Moderate/severe MR	2.602 (0.819–8.272)	0.105	0.844 (0.435–1.637)	0.616

Severe TR	3.622 (1.267–10.354)	0.016	1.401 (0.699–2.810)	0.342

Previous valvuloplasty	0.345 (0.074–1.603)	0.174	0.693 (0.358–1.339)	0.275


CI = confidence interval; HR = hazard ratio; LVEF = left ventricular ejection fraction; MV = mitral valve; NYHA = New York Heart Association; PMV = percutaneous mitral valvuloplasty; TR = tricuspid regurgitation.

**Figure 3:**
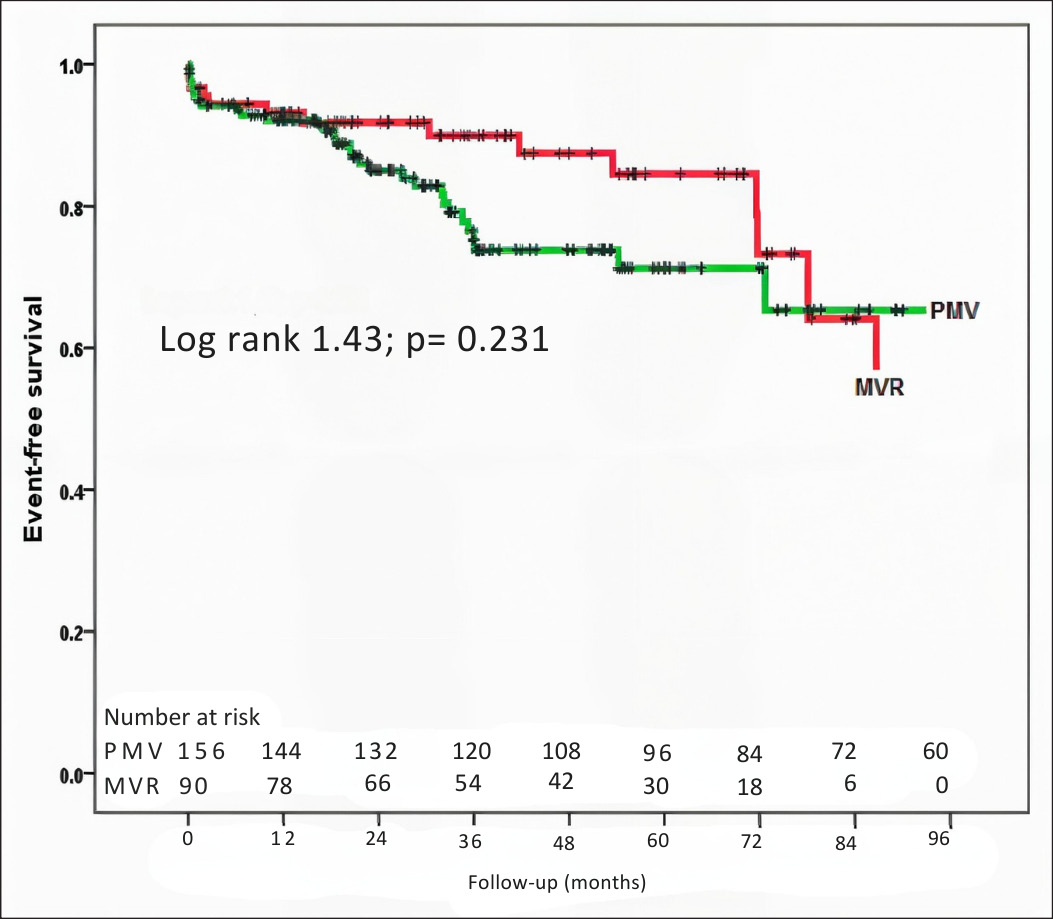
Kaplan-Meier survival curves comparing composite outcomes between patients who underwent percutaneous mitral valvuloplasty (PMV) and surgical mitral valve replacement (MVR). The selected endpoint was a composite outcome of death, repeat PMV, need for cardiac surgery, and stroke.

Unadjusted survival curves are shown in Figure 3. PMV outcomes were comparable to MVR. At 1-year follow-up, event-free survival rates were 91% for PMV patients and 92% for MVR patients (P = 0.470). After two years, there was a non-significant trend for a more favorable outcome for MVR versus PMV; 5-year event-free survival rates were 85% for MVR versus 71% for PMV (P = 0.07). After six years of intervention, event-free survival was similar between the two types of intervention, with survival rates of 75% for MVR versus 65% for PMV (p = 0.233).

**Figure 4:**
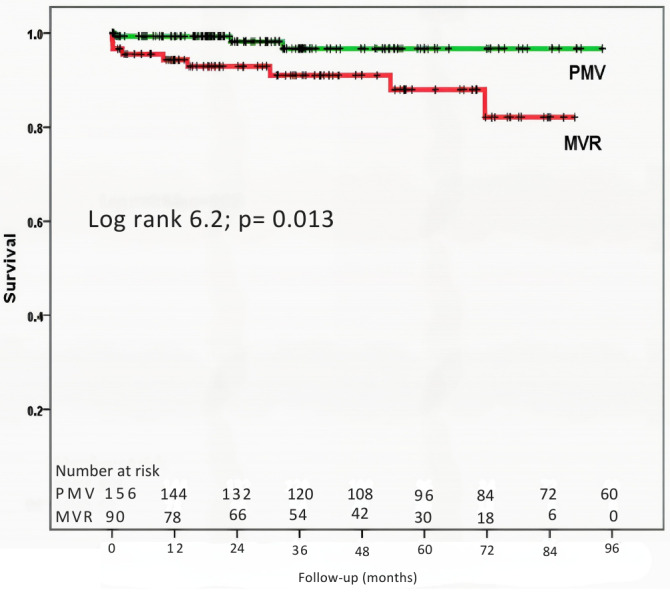
Kaplan-Meier survival curves comparing survival rates between patients who underwent percutaneous mitral valvuloplasty (PMV) and surgical mitral valve replacement (MVR). The selected endpoint was all-cause mortality.

In the Cox multivariable proportional hazards analysis, adjusted for baseline differences between groups, age and NYHA functional class emerged as independent predictors of the composite outcomes (Table 5). In terms of mortality, baseline variables reflecting the impact of MV disease, including atrial fibrillation, left ventricular ejection fraction, and age, were associated with an increased risk of death. The PMV group had a lower mortality rate compared to the surgical group (3.2% vs. 12.2%; P = 0.005) (Figure 4). However, in the multivariable proportional hazards analysis, only age and left ventricular ejection fraction remained independent predictors of mortality risk (Table 5). Notably, left ventricular ejection fraction ≤60% was associated with a greater mortality risk (hazard ratio [HR] of 5.3, 95% confidence interval [CI] of 1.4–19.4; P = 0.013) compared with the value >60%.

**Table 5:** Multivariable Cox proportional-hazards analysis for predicting death and composite outcome.


VARIABLES	ALL-CAUSE MORTALITY		COMPOSITE OUTCOME	
	
	HR (95% CI)	p-VALUE	HR (95% CI)	p-VALUE

PMV	0.351 (0.105–1.263)	0.111	1.619 (0.803–3.263)	0.226

Age (years)	1.081 (1.030–1.135)	**0.002**	1.032 (1.005–1.061)	**0.020**

NYHA class III/IV	2.519 (0.761–8.342)	0.130	2.103 (1.087–4.067)	**0.023**

Atrial fibrillation	1.867 (0.514–6.788)	0.343	0.398 (0.119–1.328)	0.134

LVEF (%)	0.933 (0.880–0.990)	**0.021**	0.959 (0.903–1.018)	0.169

Severe TR	1.995 (0.606–6.571)	0.256	0.801 (0.249–2.569)	0.708


CI = confidence interval; HR = hazard ratio; LVEF = left ventricular ejection fraction; MV = mitral valve; NYHA = New York Heart Association; PMV = percutaneous mitral valvuloplasty; TR = tricuspid regurgitation.

## Discussion

In the present study, we observed the following key findings: 1) the majority of patients who required MV intervention had mixed mitral valve disease with coexistence of MS and MR, which may have incremental pathological effects beyond those of either lesion alone; 2) patients undergoing MVR were more frequently male and tended to be older, with more evident left ventricular remodeling and higher echocardiographic scores, whereas those undergoing PMV had smaller MV area but similar pressure gradients; 3) predictors of outcomeswere older age and NYHA functional class III/IV, while predictors of mortality following either intervention were older age and lower left ventricular ejection fraction s; 4) long-term clinical outcomes were similar between surgical and percutaneous interventions over a six-year follow-up.

### Intervention strategies in rheumatic MV disease

Definitive treatment of symptomatic rheumatic MV disease is based on either surgical MVR or PMV. While MV repair is superior to MVR in select patients due to lower mortality (20), it is associated with higher rates of reoperation (16,20).

The choice of intervention depends on the pattern of rheumatic valve involvement, including the predominant valve lesion, symptoms, and hemodynamic consequences (8,9). However, mixed valve disease presents a challenge, as current noninvasive and invasive assessments have limitations in accurately defining severity. Furthermore, the optimal timing of intervention for patients with moderate mixed mitral disease remains unclear due to limited evidence.

Recent studies suggest that PMV indications have expanded to include patients with unfavorable valve anatomy, reflecting both epidemiological shifts in patient populations and advancements in interventional techniques (27,28,29,30). Our findings confirm this trend, as 63% of patients underwent percutaneous intervention. However, the expansion of PMV indications should only be considered in centers with experienced operators and immediate surgical backup to manage complications (29).

Previous studies comparing **surgical commissurotomy and PMV** in **isolated MS** have demonstrated **similar outcomes** (14,17,31). Reyes et al. (14) demonstrated that balloon valvuloplasty and open surgical commissurotomy yielded comparable initial results and low restenosis rates, maintaining good functional status for at least three years. Further studies reinforced these findings, demonstrating equivalent early hemodynamic improvements in patients with isolated MS (15). Nevertheless, in cases of concomitant MR or suboptimal valve anatomy, MVR is often preferred (8,9). For patients who failed PMV in particular, the surgical approach should be considered. In our study, 59 patients (38%) referred for PMV had undergone prior valvuloplasty, emphasizing that MV restenosis is a frequent indication for repeat intervention. For patients failing PMV, surgical intervention remains the preferred approach.

The long-term durability of valvuloplasty, whether surgical or percutaneous, in patients with extensive valve deformity remains uncertain. Late symptomatic deterioration often occurs, and reoperation rates remain high (17), even in experienced centers. Studies reported freedom from reoperation at 20 years in only 50%–60% of cases (32). A large series from Korea showed that MV repair was accomplished in 22% of patients undergoing surgery for RHD, and among them, one-third developed significant MS or MR within ten years. (19). Similarly, Bouleti et al. (33) reported that even after a successful PMV, two-thirds of patients required repeat intervention over a 20-year follow-up, with the majority undergoing MVR.

In ischemic MR, MVR has demonstrated superior durability in correcting regurgitation, potentially influencing long-term outcomes (34). Song et al. (35) found that patients with an echocardiographic score >8 and atrial fibrillation had better long-term outcomes with MVR, suggesting that early valve replacement should be considered in these cases. Another study by Kim et al. (36) compared MVR with repeat PMV in patients with mitral restenosis after previous PMV, showing that MVR provided superior long-term outcomes and was more effective than repeat PMV for up to 15 years. Kim et al. (36) also demonstrated that outcome was initially similar between groups for 40 months, but MVR had a clear advantage after a long follow-up period. In contrast, our study found no outcome differences between the two interventions. This may be due to our shorter follow-up period compared to Kim’s 15-year study.

A key consideration is whether patients should undergo MVR earlier, given that many will ultimately require a prosthetic valve in the long term (17). There are no comparative studies to answer this question definitively. The issue of repair versus replacement in rheumatic MV disease has been debated for years, with some studies reporting better survival with repair, despite its greater reoperation rate (16,20,37,38).

Although MVR improves survival in symptomatic patients, it carries substantial perioperative risks and long-term complications. Operative mortality for isolated MVR ranges from 3% to 8% in most centers (39). In the present study, the overall mortality rate was 4.9% (12 out of 246), even considering that 16% of the surgeries involved both mitral and aortic replacement. In addition, MVR frequently leads to lifelong anticoagulation and poses long-term risk of prosthesis-related complications.

### Study limitations

Our study has several limitations inherent to its retrospective design, including potential selection bias and missing data. The heterogeneous patient population included a spectrum of rheumatic MV disease, ranging from isolated pliable MS to severe predominant MR and mixed mitral disease, making direct comparisons a challenge in disease management.

Although selection bias may exist due to differences in intervention strategies, which were influenced by clinical characteristics, valve anatomy, institutional management protocols, and operator expertise. Additionally, while missing data were limited (<2% for key variables), no imputation techniques were applied. Despite these limitations, our study provides valuable insights into the outcomes of MV interventions in a real-world setting and highlights the factors associated with long-term prognosis in patients with rheumatic MV disease.

## Conclusion

In this study, including a broad spectrum of patients with rheumatic MV disease, the predictors of adverse events were age and NYHA functional class, while age and left ventricular ejection fraction were predictors of mortality following both surgical and percutaneous intervention. Notably, long-term clinical outcomes did not differ between surgical and percutaneous approaches over a follow-up period of up to six years.

These findings have critical implications for clinical decision-making, particularly in LMICs, where limited access to surgical facilities and specialized care often constrains treatment options. Given that PMV demonstrated comparable long-term outcomes to surgery, it should be prioritized in eligible patients, particularly in resource-limited settings, as it offers a less invasive, cost-effective alternative to valve replacement.

**Figure:**
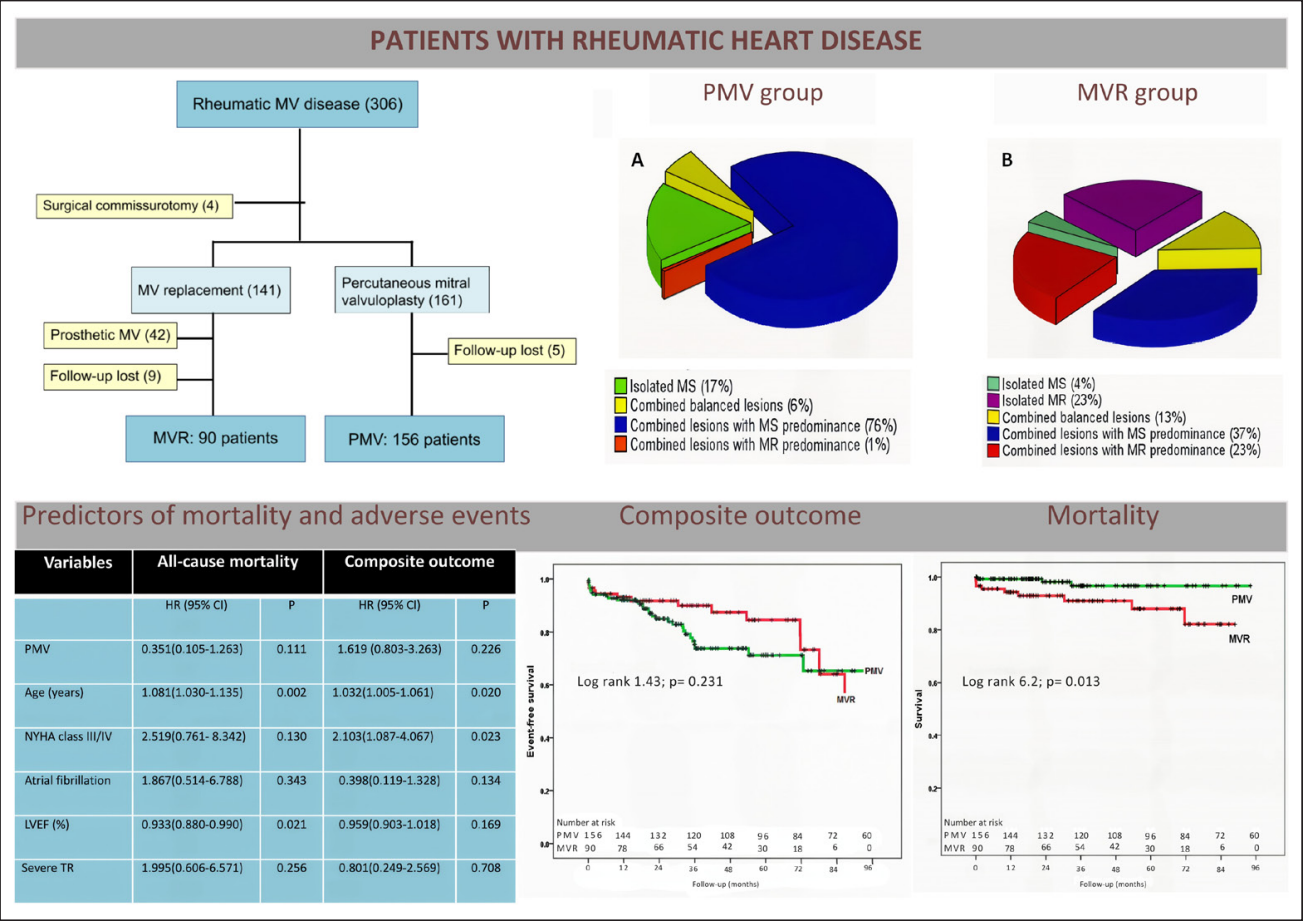
Central Figure.
